# Development of a paper strip test for colorimetric detection of urea in raw materials for animal feed

**DOI:** 10.1002/fsn3.3285

**Published:** 2023-02-28

**Authors:** Chuthamart Chanawanno, Chetbodin Sompen, Thiphol Satarpai

**Affiliations:** ^1^ Food and Feed Research Centre of Excellence, Central Laboratory CPF (Thailand) Public Company Limited Samutprakarn Thailand

**Keywords:** animal protein, fishmeal, urea adulteration, urea paper strip

## Abstract

Aiming at developing an easily implementable method for on‐site analysis to detect urea adulteration in feed ingredients, a simple and inexpensive paper strip for urea detection via colorimetric assay is herein presented. The paper strip can be simply fabricated by immobilizing urease with bromothymol blue (BTB) as a pH indicator on cellulose fiber. Upon dipping the paper strip into the target sample, the release of ammonia during the reaction between urea in the sample and urease on the paper strip causes a pH change that results in the development of a blue color, thus indicating the presence of urea. A semiquantitative detection method was developed on the basis of the color change on the paper strip, which can be detected by naked eyes and compared with a color chart made by spiking urea at concentrations varying from 0.10% to 1.0% (w/w) in animal protein and fishmeal samples. Moreover, quantitative data were obtained by taking a picture with a smartphone camera and measuring the color intensity using ImageJ software. A comparison between BTB and phenol red as pH indicators revealed that the former afforded better results in terms of resolution. Under optimal conditions, good linear responses of blue intensity were obtained in a concentration range of 0.10%–1.0% (w/w). The recovery was determined to range between 98.1% and 118.3% with a relative standard deviation of <5%. The developed paper strip assay was applied to determine urea in animal protein and fishmeal, finding good agreement with the official AOAC method (No. 967.07). The present paper strip is rapid and requires neither sophisticated devices nor skilled personnel, allowing its use by quality controllers for the routine on‐site detection of the urea adulteration of raw materials.

## Graphical abstract

Urea determination in feed ingredients based on urease‐catalyzed hydrolysis and colorimetric detection by using bromothymol blue as a pH indicator has been introduced. This design can be used both semiquantitative analysis and quantitative approach. Successful application of the urea paper strip to the determination of urea in animal protein and fish meal will be helpful for routine on‐site inspection.

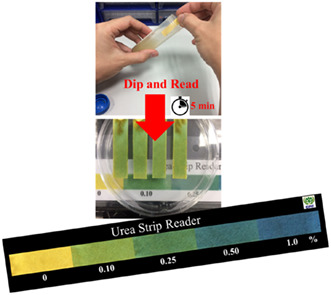

## 1 INTRODUCTION

Urea, also known as carbamide, is the major end product of nitrogen metabolism in mammals (ureotelic animals) and is produced in the liver, transported by the bloodstream to the kidneys, and excreted as urine. By contrast, the main end product of nitrogen metabolism in poultry (uricotelic animals) is uric acid; therefore, there is a significant difference in the urea content of mammal meat and poultry meat (Pibarot & Pilard, 2012). Besides, since urea occurs naturally in animals and some plants, it may be used to adulterate animal feed or its ingredients to increase the nitrogen source. Therefore, monitoring urea adulteration of feed ingredients is important to prevent the use of nonauthorized nitrogen sources. This has been traditionally conducted using spectrophotometric methods based on colorimetry (AOAC official method 967.07; AOAC, 2016). In 2014–2016, Natarajan et al. reported the investigation of urea contamination in various feeds and feed ingredients using a spectrophotometric method, which revealed that about 35.1%–40.3% of 461 samples were highly contaminated (exceeding the recommended level of 1.00%; Natarajan et al., 2018). Furthermore, liquid chromatography–electrospray ionization high‐resolution mass spectrometry (LC/ESI‐HRMS) has been used to determine urea in pet food (Pibarot & Pilard, 2012). The determination of urea is demanded not only in agroindustrial applications but also in environmental monitoring (Lambert et al., 2004; Price & Harrison, 1987), cosmetics (Bojic et al., 2008), clinical diagnostics (Ali et al., 2018), and food processes (Iida et al., 2006; Khan et al., 2015). Several analytical methods have been reported for the determination of urea, including enzymatic and nonenzymatic methods (chemical assay) and other methods such as refractive index (RI), nuclear magnetic resonance spectroscopy (NMR), Raman spectroscopy, and infrared spectroscopy (IR), which have been reviewed by Francis et al. (2002). Most of these methods have been combined with optical methods such as ultraviolet (UV)–visible photometric (Watt & Chrisp, 1954; Zawada et al., 2009), fluorimetric (Parashar et al., 2015; Zhang et al., 2014), manometric (Renny et al., 2005), and electrochemical detection (Adeloju et al., 1997; Reshetilov et al., 2015; Saeedfar et al., 2013; Velychko et al., 2016).

In this work, a paper strip based on an enzymatic reaction was developed for the semiquantitative and quantitative analysis of urea in animal protein and fishmeal via colorimetric detection. The presence of urea manifested as distinct color changes of a pH‐responsive dye that developed after the reaction between urea and urease on the paper strip. Kumar et al. previously reported a test strip based on an enzymatic reaction using phenol red (PR) as a chromogen for the semiquantitative analysis of urea in serum (Kumar et al., 2000). However, the color changes of PR are not sufficiently distinct for visual comparison with a color chart by naked eyes. Meanwhile, a urea fiber‐optic sensor using bromothymol blue (BTB) as a pH indicator was reported for the analysis of urea in fertilizers (Xie et al., 1990). Unfortunately, the sensor setup was not appropriate for on‐site inspection. To the best of our knowledge, none of these sensors has been used for urea determination in animal protein and fishmeal. In the present work, BTB and PR were studied as pH indicators, finding that the reaction on the paper strip in the presence of PR produces different shades of red, whereas BTB gives a wider range of color variations spanning from yellow to light green and blue depending on the urea concentration in the sample. To simplify the operating procedure and interpretation, a dip‐and‐read approach was used. The color on the paper strip was detected by naked eyes and compared with a color chart. The simplicity of this method renders it suitable for use by non‐laboratory‐trained staff for the quality control of feed ingredients and for on‐site inspection because it does not require complicated instrumentation. For quantitative analysis, a picture of the paper strip was taken using a smartphone camera and the color intensity was measured using the ImageJ software. The results were compared with those of the AOAC official method (nonenzymatic method) for the determination of urea in feed ingredients using a spectrophotometer.

## 2 EXPERIMENTAL

### 2.1 Chemicals and reagents

All chemicals used were of analytical reagent grade except PR, which was of laboratory grade. Urease, glycerol, sodium hydroxide (NaOH), *p*‐dimethyl amino benzaldehyde (DMAB), ethanol, hydrochloric acid, acetic acid, potassium dihydrogen phosphate, dipotassium hydrogen phosphate, potassium ferrocyanide trihydrate, and 3′, 3″‐dibromothymolsulfonphthalein (BTB) were purchased from Merck (Darmstadt, Germany). Activated charcoal was purchased from Sigma‐Aldrich (MO, USA). Phenolsulfonphthalein (PR) was purchased from Ajax Finechem Pty Ltd (NSW, Australia). Urea was purchased from Sigma‐Aldrich (Steinheim, Germany). Zinc acetate dehydrate was obtained from Loba Chemie (Mumbai, India). All stock solutions were prepared in deionized (DI) water.

A phosphate buffer solution (pH 7.0) was prepared by dissolving 3.403 g of anhydrous potassium dihydrogen phosphate and 4.355 g of anhydrous dipotassium phosphate in 900 mL of DI water. Then, the pH of the buffer solution was adjusted to 7.0 and the final volume was adjusted to 1 L with DI water.

### 2.2 Fabrication of the paper strip for urea detection

The paper strip was fabricated by cutting qualitative filter paper (Whatman® no. 3, 0.390‐μm‐thick) into 1.0 cm × 8.5 cm pieces using the knife‐cutting method. Urease (0.1 g; specific activity of 11 U/mg solid) was dissolved in 20 mL of DI water and mixed thoroughly in a mortar (Solution A). The chromogen (BTB or PR; 60 mg) was dissolved in 0.96 mL of 0.1 N NaOH followed by the addition of 20 mL of DI water, and the mixture was mixed well (Solution B). After mixing solutions A and B, 4.4 mL of glycerol was added, and the mixture was sonicated in an ultrasonic bath for 20 min to promote the dissolution. The final concentration of urease was about 24 U/mL. Subsequently, the solution was successively filtered using filter paper no. 3. The paper was completely submerged in the reagent and allowed to dry in a fume hood at ambient temperature. The strip was kept in an amber plastic bag and stored below 4°C in a refrigerator until use. To prepare a blank paper strip without urease, only solution B was used in a final volume of 40 mL. To avoid lot‐to‐lot variation, the pH was fixed before at basic condition to fix the color of the paper strip. A final NaOH concentration of 0.002 N was used to preserve the protein structure of the enzyme.

### 2.3 Paper strip test for urea colorimetric detection

**FIGURE 1:**
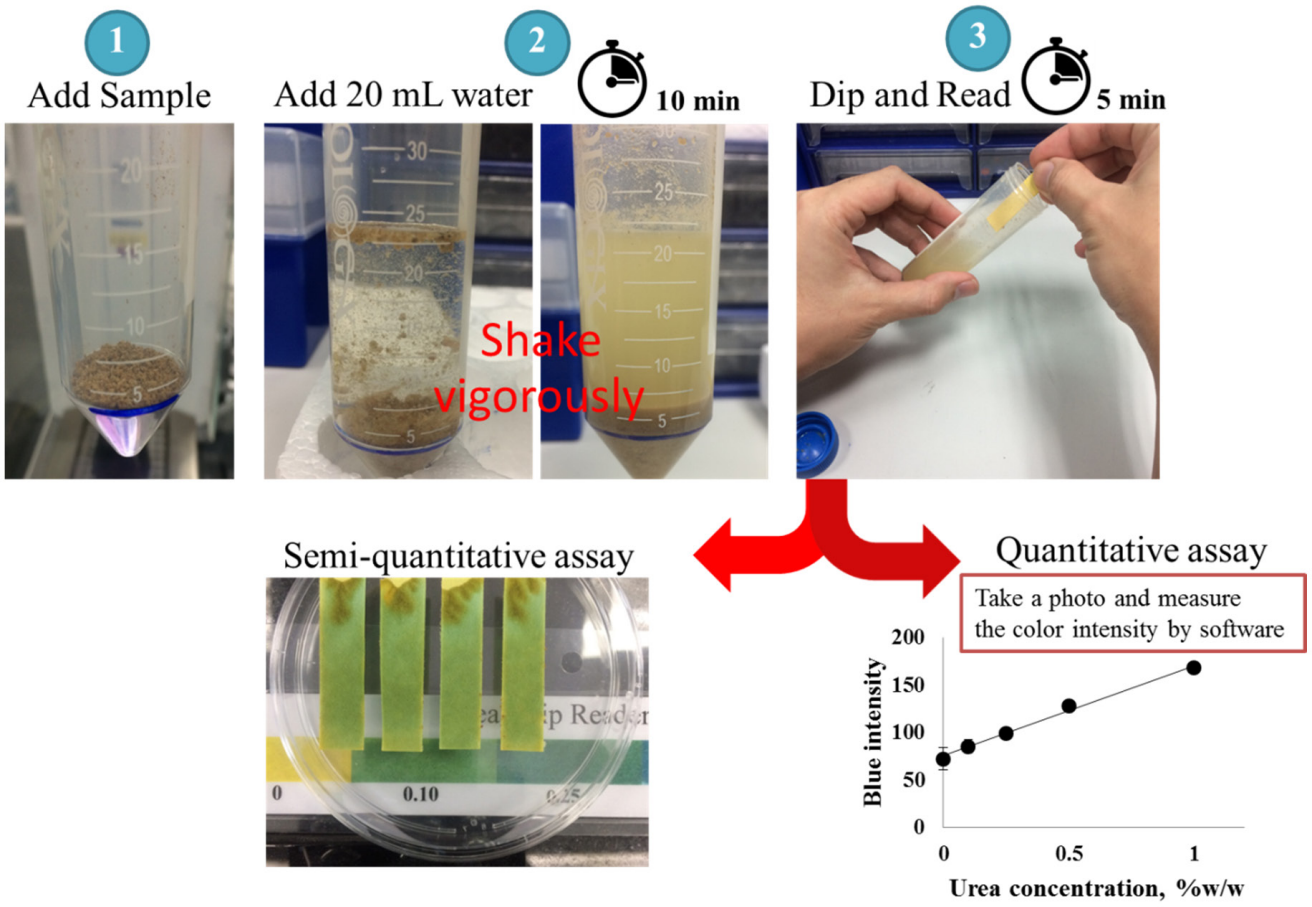
Schematic of the procedure for urea detection using the paper strip assay.

A schematic of the procedure for the colorimetric detection of urea using the developed paper strip is depicted in Figure 1. Briefly, a test sample was transferred into a test tube up to a level of about 2 g and dissolved in 20 mL of distilled water. The mixture was shaken briefly. The tube was kept at room temperature for 10 min to allow sedimentation. Then, the paper strip was dipped into the extracted solution and the color was read at 5 min after dipping. For the semiquantitative analysis, the paper strips were put onto a plate and the color was read by comparing with a urea chart. The color charts or urea strip readers of animal protein (black) and fishmeal (white) in a urea concentration range of 0%–1.0% (w/w) are shown in Figure 5. The blank strip was dipped into the extracted solution to determine the pH of the native extracted sample. In the absence of urea, the color of the blank strip should be the same as that of the test strip. The paper strip for urea detection was appropriately designed for use at the native pH of the extracted animal protein and fishmeal samples. However, if the color of the blank strip changes, subtraction of the mean intensity would be required.

For the quantitative analysis, a mobile phone (iPhone 5s, Apple Inc.) was placed on a stand and the camera factors were fixed using the camera application in auto mode. The color change of the paper strip was recorded by taking a photograph, and the blue intensity was measured using the image processing software ImageJ (NIH, USA).

### 2.4 Spectrocolorimetric method for urea analysis in animal feeds and their ingredients

For comparative purposes, the official AOAC method (No. 967.07) based on the yellow–green color produced by DMAB was applied (AOAC, 2016). In brief, 5.00 g of ground sample was transferred into a 250‐mL volumetric flask, and 1.5 g of charcoal, 5 mL of 1.0 M zinc acetate, 5 mL of 0.25 M potassium ferrocyanide, and 100 mL DI water were added. After the mixture was shaken for 30 min, the volume was adjusted to 250 mL with DI water and filtered. An aliquot of 5 mL was pipetted into a test tube and mixed with 5 mL of 0.1 M DMAB. The solution was shaken and kept at room temperature for 10 min to allow sedimentation before measurement. The spectrophotometric measurements were performed on a UV‐1800 spectrophotometer (Shimadzu Co., Kyoto, Japan) at 420 nm against a blank (instead of 5 mL of aliquot sample or 5 mL of buffer). A calibration curve was constructed by pipetting 5 mL of the working standard solution with a concentration ranging from 0.2 to 4.0 mg/5 mL and mixing it with 5 mL of 0.1 M DMAB. The concentration of urea was calculated using Equation (1).

(1) $$\text{Urea}\%\left(w/w\right)=\left[\left({\text{Conc}}_{\text{sample}}-{\text{Conc}}_{\text{reagent blank}}\right)\times 250\right]/\text{weight}\;\left(g\right)$$

## 3 RESULTS AND DISCUSSION

### 3.1 Comparative study on urea determination using a spot test and the paper strip method

The colorimetric reaction was tested using a spot test and the newly developed paper strip method for the detection of urea in animal feed and its raw materials. The spot test procedure (Islam et al., 2016; National Dairy Development Board (NDDB), 2013) was also used to fabricate the paper strip for urea detection. In brief, 2.00 g of the sample was transferred into a test tube and dissolved in 20 mL of DI water. The solution was shaken thoroughly and kept at room temperature for 10 min before sampling. In the spot test, the color change was successfully detected in the spot plate after adding eight drops of sample test, three drops of 0.1% BTB, and four drops of 0.4% urease solution. The color changed as a result of the pH increase caused by the release of ammonia from the urease‐catalyzed hydrolysis of urea in solution, as described by Equation (2).

(2) $${\mathrm{CH}}_{4}N_{2}O+H_{2}O\;\overset{\text{Urease}}{\to }\;2{\mathrm{NH}}_{3}+{\mathrm{CO}}_{2}$$

**FIGURE 2:**
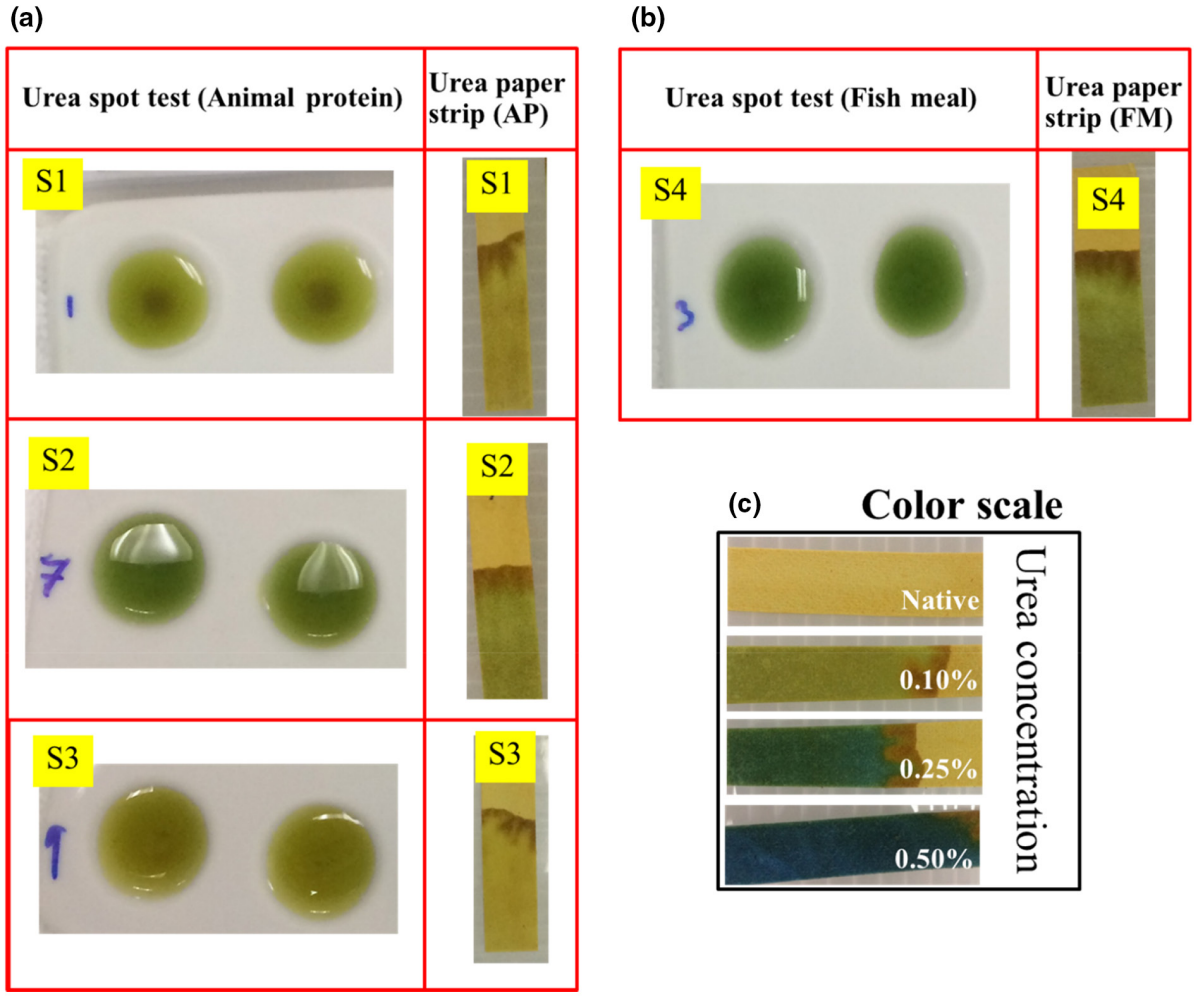
Colorimetric results of a spot test and the paper strip method for urea determination in (a) animal protein and (b) fishmeal. (c) Image showing the color scale of a urea standard solution.

The paper strip was preimmobilized with urease and the pH indicator on the cellulose fiber of the paper strip, in which the reaction occurred according to Equation (2). As shown in Figure 2c, which displays the correlation between the color change and the amount of urea, the color of the paper strip changed from green to blue when the concentration of urea increased from 0.10% to 0.50% (w/w). The detection limit was 0.10% (w/w). S. Islam et al. reported a detection limit of 0.25% in a spot test for the determination of urea in raw materials of feed using 0.1% of BTB as a chromogen (Islam et al., 2016). The spot test and the paper strip test for animal protein and fishmeal are compared in Figure 2a,b, respectively. Similar results were obtained, demonstrating the good performance of the paper strip. Moreover, the paper strip has the advantage of the lack of interference from a turbid solution or the presence of suspended solids on the bottom of the spot plate. Urea concentrations of 0.10% (w/w) were found in Sample no. 2 and 4 (animal protein and fishmeal, respectively).

### 3.2 Effect of using different pH indicators on the color change in the paper strip test

**FIGURE 3:**
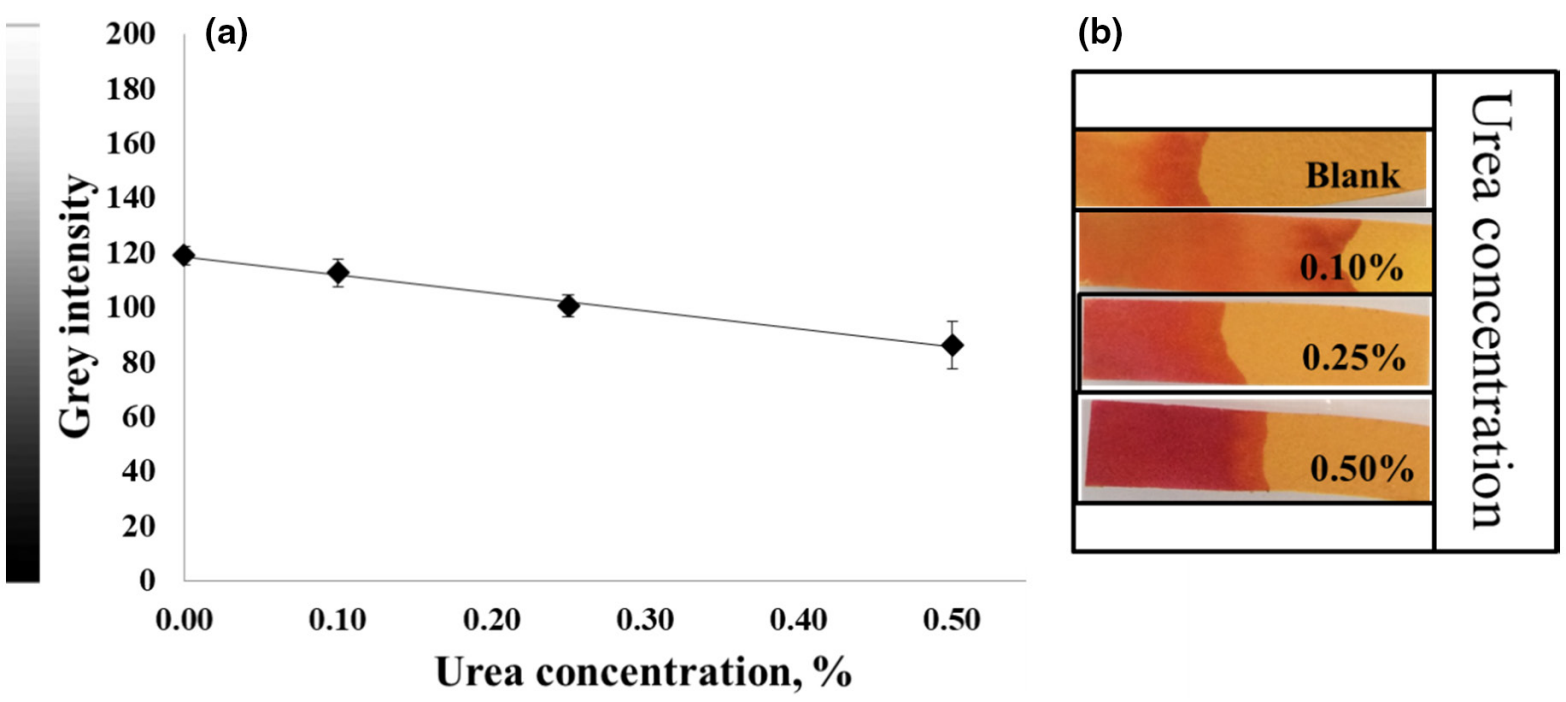
(a) Calibration plot of the gray intensity value as a function of the urea concentration in the range of 0.10%–0.50% (w/w). The linear equation was *y* = −76.45*x* + 117.63 and the correlation coefficient was 0.9798. (b) Photograph of the paper strip with phenol red as a pH indicator.

Different color shades were obtained when using PR or BTB in the paper strip test for the determination of urea. The color ranged from light pink to deep magenta for PR (pKa = 7.9) (Figure 3b), whereas BTB (pKa = 7.1) produced a color change from light green to blue depending on the urea concentration (Figure 5). In addition, the linearity range for animal protein samples spiked with urea was 0.10%–0.50% (w/w) with a correlation coefficient of 0.9798 in gray intensity (Figure 3a) for PR. In contrast, BTB provided a linearity range of about 0.10%–1.0% (w/w), as shown in Figure 5. BTB afforded a clearer color discrimination by naked eyes and a wider linearity range than PR. Thus, BTB was proved to be an effective pH indicator to detect urea in feed ingredients and was accordingly selected as a pH indicator for subsequent experiments. Nevertheless, it should be noted that Kumar et al. (2000) used PR as a chromogen for the determination of urea in serum in a concentration range of 0.15–2.00 g/L, indicating that PR can be used for urea detection at low concentrations.

**FIGURE 4:**
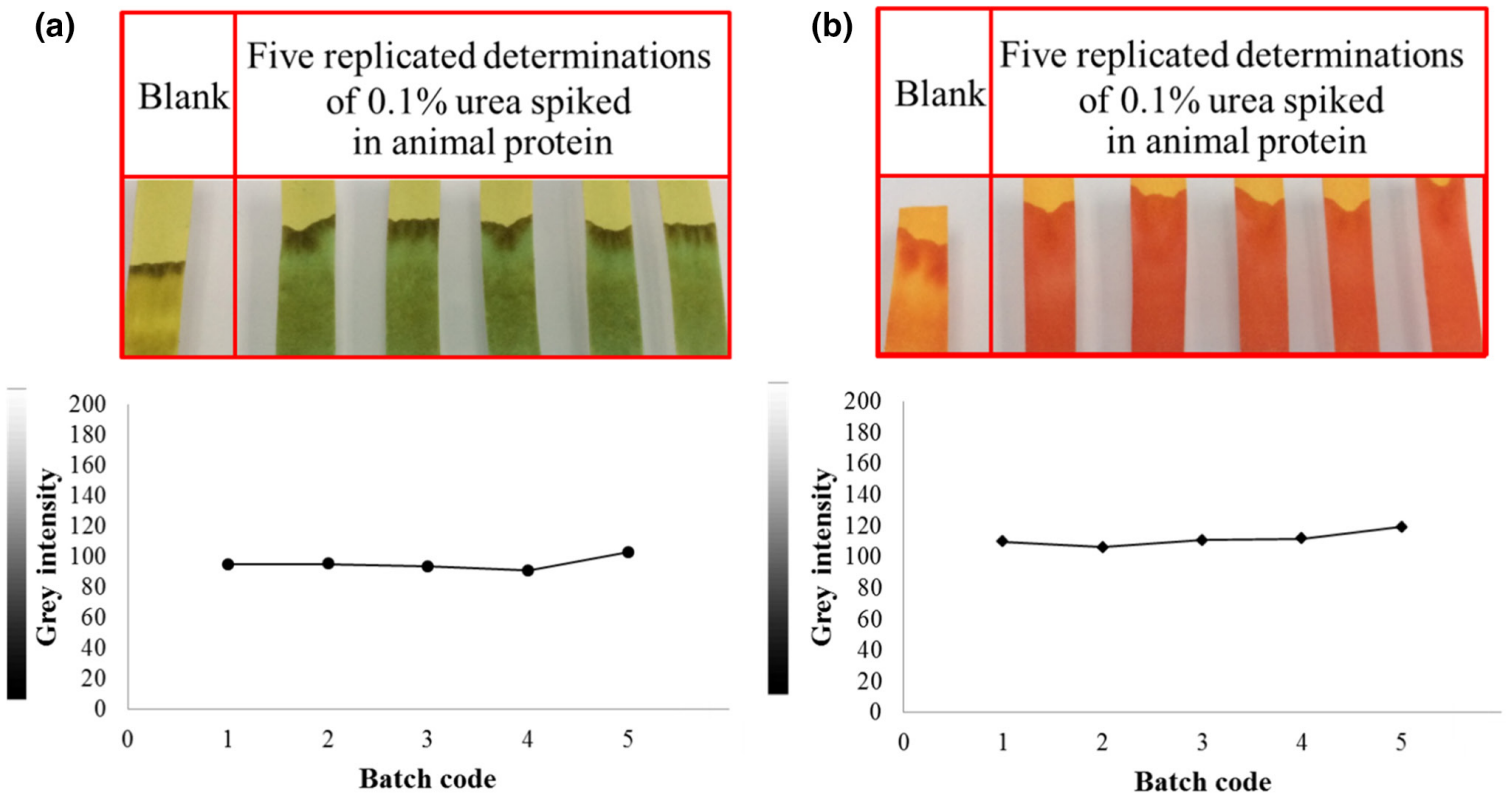
Reproducibility of the paper strip tested at a urea concentration of 0.10% (w/w) in animal protein with (a) bromothymol blue and (b) phenol red as pH indicator (*n* = 5).

The reproducibility between different batches of strips was also studied, and gray intensity measurements using BTB and PR were compared. The relative standard deviation of BTB and PR was 4.7% and 4.2%, respectively (*n* = 5). As shown in Figure 4a,b, the reproducibility of both pH indicators was satisfactory.

### 3.3 Effect of detection time on the color intensity and resolution of the paper strip for urea detection

To find an optimal detection time after dipping the paper strip in the sample solution, the blue intensity was recorded against the reading time. The pH change was monitored using BTB within 30 s at room temperature. As shown in Figure 6, the blue intensity increased during the initial 3 min before reaching a constant value. A distinct resolution between urea concentrations of 0.25% and 0.50% (w/w) was observed between 4 and 5 min. Therefore, 5 min was selected as the optimal detection time for the paper strip.

### 3.4 Analytical performance

**FIGURE 5:**
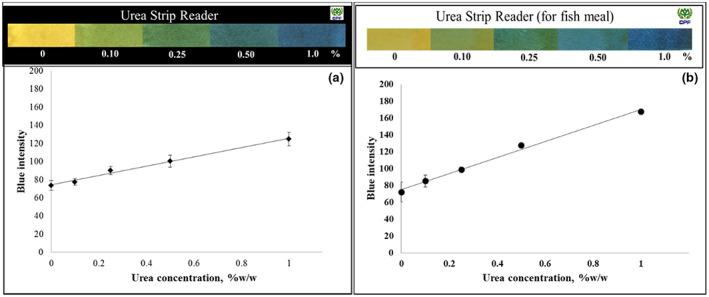
Calibration plot of the blue intensity value as a function of the concentration of urea spiked in (a) animal protein and (b) fishmeal.

Next, the analytical features of the developed method for the quantitative analysis of urea, including the linear range of the calibration curve, precision, and accuracy, were investigated. The gray and RGB intensity were plotted against the urea concentration to compare the sensitivity response. The blue intensity analysis showed the best sensitivity; thus, the blue intensity was selected as the measurement parameter. The linear equations for animal protein and fishmeal were *y* = 51.41*x* + 74.3 and *y* = 95.07*x* + 75.1, respectively. Good linearity was observed for the calibration curves in the concentration range of 0.10%–1.0% (w/w) with a correlation coefficient of 0.9914 for animal protein (Figure 5a) and 0.9930 for fishmeal (Figure 5b). The precision of the developed method was below 10% RSD for animal protein and fishmeal. The accuracy was evaluated by spiking animal protein and fishmeal with urea at concentrations between 0.10% and 1.0% (w/w). Table 1 summarizes the results obtained using the paper strip and the official method AOAC 967.07. A recovery ranging between 98.1% and 118.3% was observed for the developed method, which was in good agreement with that of the spectrophotometric technique.

**TABLE 1:** Urea concentration found in animal protein (S5 and S6) and fishmeal (S7 and S8) samples and analytical recovery using the paper strip test and the AOAC spectrophotometric method (*n* = 3).

Sample	Urea strip test		AOAC official method 967.07	
	Urea, % (w/w)	Recovery, %	Urea, % (w/w)	Recovery, %
S5	0.042 ± 0.009	—	0.061 ± 0.05	—
S6	0.010 ± 0.001	—	0.024 ± 0.07	—
S7	0.044 ± 0.007	—	0.045 ± 0.01	—
S8	0.045 ± 0.01	—	0.052 ± 0.03	—
S6 spiked 0.10% (w/w) urea	0.12 ± 0.02	118.3	0.12 ± 0.05	96.0
S6 spiked 0.25% (w/w) urea	0.29 ± 0.08	115.2	0.27 ± 0.03	98.4
S6 spiked 0.50% (w/w) urea	0.52 ± 0.05	103.4	0.62 ± 0.08	119.2
S6 spiked 1.0% (w/w) urea	0.98 ± 0.11	98.1	1.20 ± 0.20	117.6

### 3.5 Determination of urea in real samples

The urea concentration in animal protein was determined using the paper strip method and the AOAC spectrophotometric method. As shown in Table 1, the concentration of urea in these samples was determined to be 0.01%–0.04% (w/w) using the paper strip assay. No effect of interfering substances in real samples on the color of the paper strip was observed. The two methods showed satisfactory correlation coefficients of 0.9944. A paired *t*‐test revealed no significant statistical difference between the paper strip method and the official method (*t*_stat_ = 1.59 and *t*_crit_ = 2.36 at *p* = .05), which demonstrates that the paper strip can be used as a reliable tool for urea determination.

**FIGURE 6:**
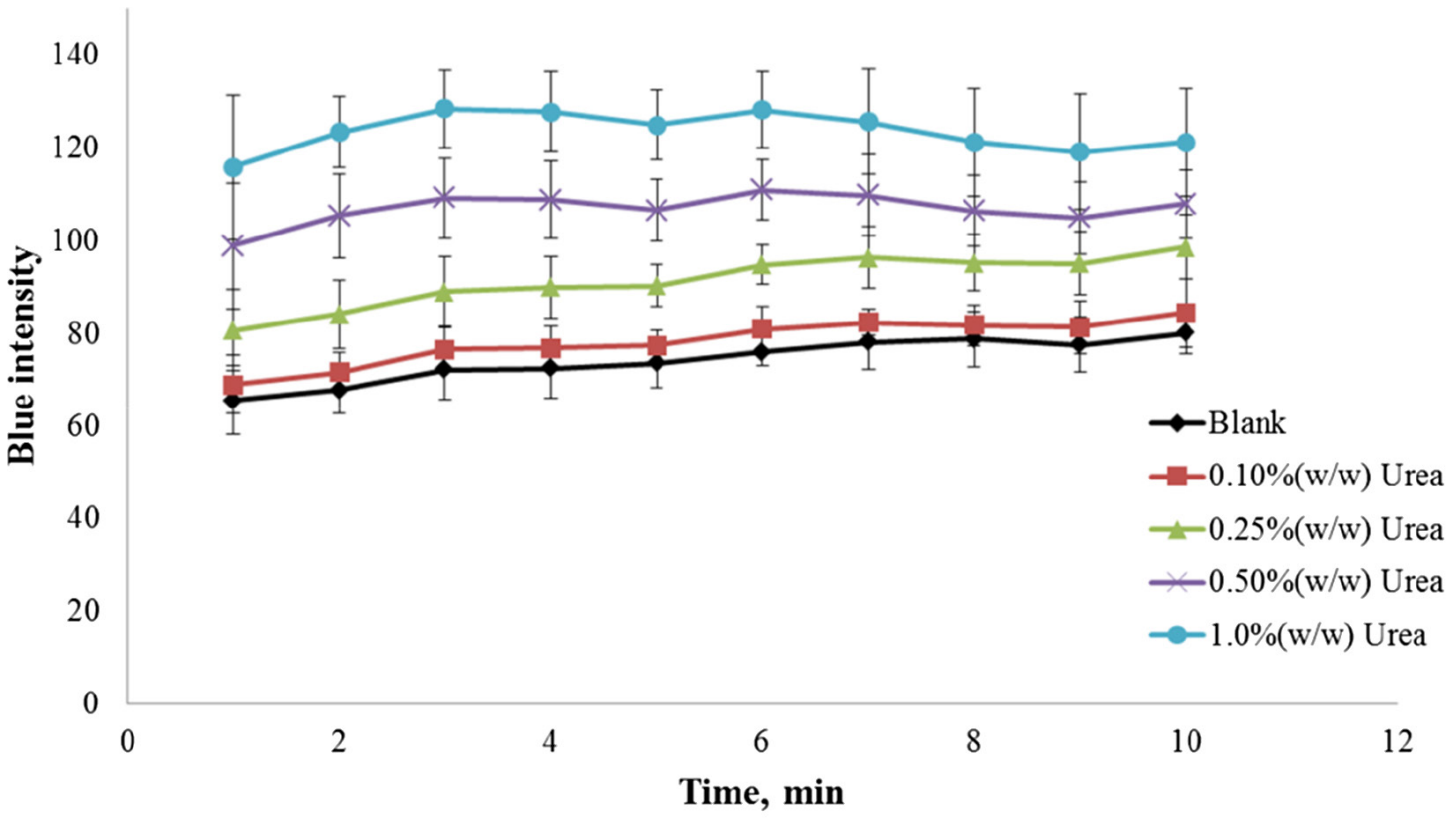
Effect of the reading time on the colorimetric detection of urea using the paper strip in the urea concentration range of 0.10%–1.0% (w/w).

To evaluate the analytical capability of the paper strip method as a routine screening test, a series of blind materials of animal protein and fishmeal were prepared by spiking a urea standard solution. The assigned value categories used in the analysis were determined by calculating the mean values obtained using the spectrophotometric technique. Twelve participants from our branch laboratories in Thailand were provided with the blind materials and conducted our paper strip test. Tables 2 and 3 show the results of urea determination in animal protein and fishmeal, respectively. The semiquantitative assay using the paper strip afforded an accuracy of 95% ± 9.0% and 88% ± 18% for animal protein and fishmeal, respectively. The mean values obtained from all participants were compared with the assigned values (mean values minus assigned values). For the animal protein samples, the mean of these differences was −0.017 and −0.021 at urea concentrations of 0.10% and 0.50% (w/w), respectively. The corresponding values for the fishmeal samples were 0.06 and −0.17 at urea concentrations of 0.50% and 1.0% (w/w), respectively. However, these bias results were mainly due to misreading of the standard color chart because the color changes of the paper strip were maintained relative to the original urea concentration. Some of the results were not consistent probably due to the reading time. As shown in Figure 6, reading times longer than 5 min resulted in lower resolution and potential overlapping with lower urea concentrations. The relative standard deviation between participants was determined to be 9.5% and 20.4% for animal protein and fishmeal, respectively.

**TABLE 2:** Comparison of various assigned values of spiked urea concentrations of blind samples (animal protein) and values provided by participants using the paper strip test.

BM code	Assigned value, % (w/w)	Participant											
		1	2	3	4	5	6	7	8	9	10	11	12
14	0.10	0.10	0.10	0.10	0.00	0.10	0.10	0.10	0.10	0.10	0.10	0.00	0.10
15	0.50	0.50	0.25	0.50	0.50	0.50	0.50	0.50	0.50	0.50	0.50	0.50	0.50
16	0	0	0	0	0	0	0	0	0	0	0	0	0
17	0.25	0.25	0.25	0.25	0.25	0.25	0.25	0.25	0.25	0.25	0.25	0.25	0.25
18	1.0	1.00	1.00	1.00	1.00	1.00	1.00	1.00	1.00	1.00	1.00	1.00	1.00
Trueness, %		100	80	100	80	100	100	100	100	100	100	80	100
Mean	95												
SD	9.0												
RSD, %	9.5												

Abbreviations: BM, blind materials; RSD, relative standard deviation; SD, standard deviation.

**TABLE 3:** Comparison of various assigned values of spiked urea concentrations of blind samples (fishmeal) and values provided by participants using the paper strip test.

BM code	Assigned value, % (w/w)	Participant											
		1	2	3	4	5	6	7	8	9	10	11	12
14	0	0	0	0	0	0	0	0	0	0	0	0	0
15	0.25	0.25	0.25	0.25	0.25	0.25	0.25	0.25	0.25	0.25	0.25	0.25	0.25
16	0.10	0.10	0.10	0.10	0.10	0.10	0.10	0.10	0.10	0.10	0.10	0.10	0.10
17	1.0	1.00	0.50	1.00	0.50	1.00	1.00	1.00	1.00	1.00	1.00	0.50	0.50
18	0.50	0.50	1.00	0.50	1.00	0.50	0.50	0.50	0.50	0.50	0.50	0.25	0.50
Trueness, %		100	60	100	60	100	100	100	100	100	100	60	80
Mean	88												
SD	18.0												
RSD, %	20.4												

Abbreviations: BM, blind materials; RSD, relative standard deviation; SD, standard deviation.

## 4 CONCLUSIONS

A simple, cost‐effective, and scalable paper strip assay for urea determination in feed ingredients was developed on the basis of the urease‐catalyzed hydrolysis of urea and colorimetric detection using BTB as a pH indicator. Using a simple dip‐and‐read method, the paper strip can be used to detect urea in contaminated raw materials within a short time and without requiring sophisticated devices or skilled personnel; therefore, it can be used by quality controllers for field screening using a well‐established semiquantitative analysis. The paper strip can be used in the urea concentration range of 0.10%–1.0% (w/w). A quantitative approach was also developed by capturing images with a smartphone camera and measuring the color intensity of the paper using the ImageJ software. The developed method demonstrated good recoveries and reproducibility for urea analysis and showed good agreement with the official method. Therefore, the paper strip for the determination of urea in animal protein and fishmeal could be used for routine on‐site inspection.

## FUNDING INFORMATION

This study was financially supported by CPF Co., Ltd. (Thailand).

## CONFLICT OF INTEREST STATEMENT

The authors declare that they have no competing interests.

## Data Availability

Research data have been provided in the manuscript. The data that support the findings of this study are available upon request from the corresponding author.
